# Mechanistic Insights and Therapeutic Potentials of Ubiquitin‐Proteasome System in Non‐Small Cell Lung Cancer

**DOI:** 10.1111/cpr.70050

**Published:** 2025-05-01

**Authors:** Guangyao Zhou, Jiaxiong Tan, Pengpeng Zhang, Zhaokai Zhou, Lianmin Zhang, Zhenfa Zhang

**Affiliations:** ^1^ Department of Lung Cancer, Tianjin Lung Cancer Center, National Clinical Research Center for Cancer, Key Laboratory of Cancer Prevention and Therapy, Tianjin's Clinical Research Center for Cancer Tianjin Medical University Cancer Institute and Hospital Tianjin China; ^2^ National Clinical Research Center for Cancer, Key Laboratory of Cancer Prevention and Therapy, Tianjin's Clinical Research Center for Cancer Tianjin Medical University Cancer Institute and Hospital Tianjin China; ^3^ Department of Urology The First Affiliated Hospital of Zhengzhou University Zhengzhou Henan China

**Keywords:** cancer therapy, NSCLC, proteasome inhibition, targeted therapy, ubiquitination

## Abstract

Non‐small cell lung cancer (NSCLC) remains a leading cause of cancer mortality. Despite advancements in gene targeted therapies and immunotherapies, high heterogeneity contributes to limited efficacy and therapeutic resistance. Ubiquitination, a crucial post‐translational modification that regulates protein stability and degradation, plays a significant role in cancer pathogenesis by influencing key oncogenic pathways and tumour progression. This review systematically explores the ubiquitin‐proteasome system (UPS) and its potential as a therapeutic target for NSCLC. We highlight recent preclinical and clinical studies focusing on ubiquitination‐related biomarkers, drug targets and emerging therapies like proteasome inhibitors and Proteolysis‐targeting chimeras (PROTACs). By exploring the impact of the UPS on tumour biology, the progression of NSCLC and its response to therapy, we aim to underscore the potential of targeting the ubiquitination‐deubiquitination system as a complementary or synergistic approach to existing therapeutic strategies in NSCLC, thereby enhancing patient outcomes and overcoming treatment resistance.

## Graphical abstract

Lung cancer development involves mechanisms like reduced EGFR/EGF ubiquitination driving cancer cell behaviors and immune evasion via loss of PD‐1/PD‐L1 ubiquitination. Balancing ubiquitination and deubiquitination may be a potential therapeutic strategy.

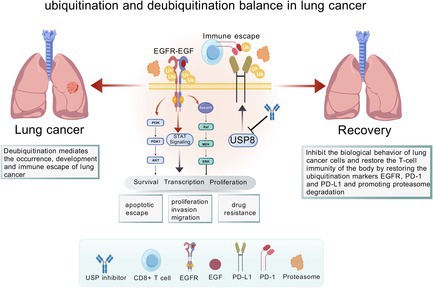

**Abbreviations**
ABLIM1: Actin‐binding LIM protein 1
ACTN4: Alpha‐actinin‐4
ALK: Anaplastic lymphoma kinase
ATM: Ataxia telangiectasia mutated
CCND1: Cyclin D1
CUL4: Cullin 4
DUBs: deubiquitinating enzymes
E1: Ubiquitin‐activating enzyme
E2: Ubiquitin‐conjugating enzyme
E3: Ubiquitin ligase
EGFR: epidermal growth factor receptor
ERK: Extracellular signal‐regulated Kinase
FBXO32: F‐box protein 32
FDA: Food and Drug Administration
FOXO6: Forkhead box O6
Hippo: Hippo signalling pathway
JAK/STAT: Janus kinase/signal transducer and activator of transcription
KEAP1: Kelch‐like ECH‐associated protein 1
LATS2: large tumour suppressor kinase 2
MDM2: Mouse double minute 2
MIB2: Mindbomb E3 ubiquitin protein ligase 2
mTORC2: mammalian target of rapamycin complex 2
NF‐κB: Nuclear factor‐kappa B
NSCLC: non‐small cell lung cancer
OTUD7B: OTU deubiquitinase 7B
P53: Tumour Protein 53
PHF23: Plant homeodomain finger protein 23
PHPT1: Phosphohistidine phosphatase 1
PROTACs: Proteolysis Targeting Chimeras
PTPN23: Protein tyrosine phosphatase, non‐receptor type 23
RBM38: RNA binding motif protein 38
SCLC: Small cell lung cancer
TGF‐β: Transforming growth factor beta
TP53: Tumour Protein 53
TRIM21: Tripartite motif‐containing 21
TβR‐1: Transforming Growth Factor Beta receptor I
UPS: Ubiquitin‐proteasome system
USP1: Ubiquitin‐specific Peptidase 1
USP10: Ubiquitin‐specific Peptidase 10
USP12: Ubiquitin‐specific Peptidase 12
USP14: Ubiquitin‐specific Peptidase 14
USP15: Ubiquitin‐specific Peptidase 15
USP2: Ubiquitin‐specific Peptidase 2
USP22: Ubiquitin‐specific Peptidase 22
USP24: Ubiquitin‐specific Peptidase 24
USP3: Ubiquitin‐specific Peptidase 3
USP34: Ubiquitin‐specific Peptidase 34
USP36: Ubiquitin‐specific Peptidase 36
USP37: Ubiquitin‐specific Peptidase 37
USP4: Ubiquitin‐specific Peptidase 4
USP49: Ubiquitin‐specific Peptidase 49
USP5: Ubiquitin‐specific Peptidase 5
USP51: Ubiquitin‐specific Peptidase 51
USP7: Ubiquitin‐specific Peptidase 7
WDR4: WD repeat domain 4

## 1 Introduction

Lung cancer remains a leading cause of cancer‐related mortality globally, with clinical strategies dictated by its pathological subtypes (NSCLC [85%] and SCLC) and molecular heterogeneity [1]. While surgery confers curative potential in early‐stage NSCLC, advanced cases require systemic therapies such as targeted agents and immunotherapy. EGFR/ALK‐targeted therapies achieve > 50% 5‐year survival in molecularly selected populations, yet 15%–20% of NSCLC patients lack actionable mutations and SCLC remains target‐poor with inherent/acquired resistance [2]. Despite immunotherapy's paradigm‐shifting impact, NSCLC patients exhibit only 20% 5‐year survival post‐treatment, reflecting challenges in sustaining immune responses amidst tumour microenvironment heterogeneity [3, 4]. These persistent limitations highlight the need for novel therapeutic targets beyond current strategies.

The ubiquitin‐proteasome system (UPS) emerges as a pivotal regulatory network governing oncogenic processes through dynamic post‐translational modifications. Ubiquitination—mediated by an enzymatic cascade involving E1 activating, E2 conjugating and E3 ligase enzymes—precisely controls protein degradation, functional modulation and subcellular localization [5, 6]. Dysregulation of this system disrupts critical signalling pathways including NF‐κB, TGF‐β, JAK/STAT, ERK and Hippo, driving malignant progression. For instance, ABLIM1‐mediated IκBα ubiquitination activates NF‐κB signalling to accelerate colorectal cancer metastasis [7], while PHF23 stabilises ACTN4 via impaired ubiquitination, promoting ERK pathway hyperactivation in NSCLC [8, 9]. Counterbalancing these effects, deubiquitinating enzymes (DUBs) such as USP family members exhibit dual regulatory roles: stabilising oncoproteins (e.g., ID proteins via USP1, TGF‐β receptors via USP4/15) or tumour suppressors (e.g., p53 via USP7) through ubiquitin chain removal [10, 11, 12, 13, 14]. The delicate equilibrium between ubiquitination and deubiquitination thus constitutes a fundamental yet underexplored axis in cancer biology [15].

Recent therapeutic advancements targeting the UPS fall into three strategic categories. First, direct degradation platforms like proteasome inhibitors (e.g., bortezomib) and proteolysis‐targeting chimeras (PROTACs) demonstrate clinical promise, with PROTACs utilising heterobifunctional molecules to recruit E3 ligases for selective protein degradation [16, 17]. Second, indirect modulation strategies employ pharmacological agents or natural compounds (e.g., curcumin analogues) to enhance ubiquitination activity via cAMP‐PKA/MAPK pathway regulation [18]. Third, multi‐omics integration enables ubiquitination‐based molecular subtyping and prognostic modelling in lung adenocarcinoma, paving the way for precision therapeutics [18]. Despite these innovations, current research remains compartmentalised, lacking systemic exploration of cross‐pathway interactions and robust translational validation.

This review explores UPS dysregulation in lung carcinogenesis, analyses therapeutic advances in ubiquitination targeting and proposes strategies to circumvent treatment resistance. By mapping crosstalk between ubiquitination networks and oncogenic pathways, we establish a framework for next‐generation drug development. Our analysis synthesises current evidence while highlighting critical knowledge gaps, advocating integrated strategies to refine precision oncology.

## 2 The Dynamic Balance Between Ubiquitination and Deubiquitination in NSCLC Pathogenesis

The malignant progression of non‐small cell lung cancer (NSCLC) is critically regulated by the ubiquitin‐proteasome pathway (UPP), which dynamically controls protein stability to coordinate interactions amongst driver gene mutations, immune evasion and cancer stem cell properties. As a central oncogenic driver, EGFR hyperactivation and therapeutic resistance can be mechanistically dissected through ubiquitination regulation: The WDR4‐Cul4 complex promotes tumorigenesis by inhibiting PTPN23‐mediated EGFR degradation, while the miR‐4487/USP37 axis and USP22 serve as positive regulators of EGFR ubiquitination and negative stabilisers, respectively, offering dual therapeutic targets to overcome EGFR‐TKI resistance [19, 20, 21]. Notably, the discovery of the PHPT1/FBXO32 node reveals the broad regulatory role of ubiquitination beyond EGFR signalling—FBXO32‐mediated PHPT1 degradation simultaneously suppresses proliferation and enhances EGFR inhibitor sensitivity [22]. These findings suggest that targeting ubiquitination nodes enables multidimensional modulation of EGFR signalling.

**FIGURE 1:**
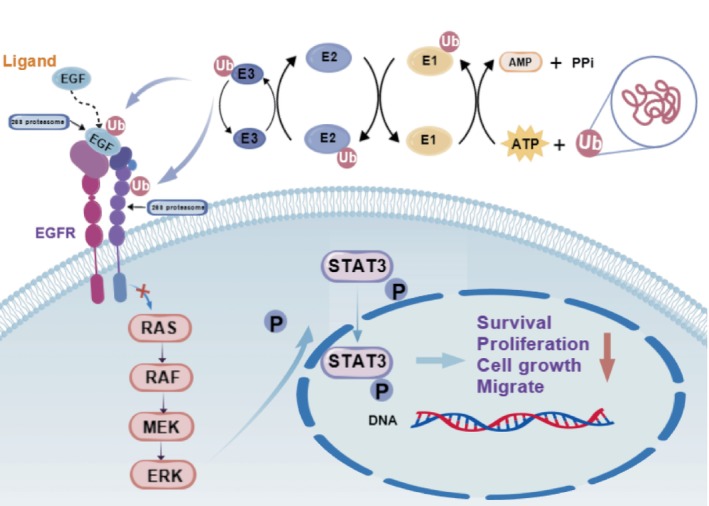
Ubiquitination‐mediated degradation of EGFR‐EGF complex and inhibition of downstream signalling in EGFR‐mutated cancer. This figure illustrates the process by which the ubiquitination system (E1‐E2‐E3) is activated to tag target proteins EGFR and GRF with ubiquitin. This tagging directs the EGFR–EGF complex to proteasomal degradation, thereby blocking downstream signalling pathways, such as the RAS–RAF–MEK signalling pathway, within cancer cells and inhibiting a variety of biological behaviours of cancer cells.

In KRAS‐mutant NSCLC, the ubiquitination network sustains tumour metabolic reprogramming through multiple mechanisms. USP5 stabilises the autophagy regulator Beclin1 to promote p53 degradation, while the RNF185/TRIM21‐TRIM4 axis enhances TPL2‐driven invasion by reducing its ubiquitination [23, 24]. OTUD7B, a critical modulator of mTORC2 complexes, demonstrates significant suppression of KRAS‐driven tumour growth upon knockout [25], providing a theoretical foundation for developing selective deubiquitinase inhibitors. These mechanisms illustrate that KRAS mutations are not isolated events but functionally coupled with autophagy and metabolic pathways via ubiquitination networks. In short, activation of the ubiquitination system not only promotes the ubiquitin labelling of the EGFR‐EGF complex for proteasomal degradation but also targets the critical signalling pathways, such as the RAS–RAF–MEK signalling pathway, thereby inhibiting the biological behaviour of EGFR‐mutated or EGFR‐TKI‐resistant lung cancer cells through dual mechanisms. (Figure 1).

Ubiquitination modifications of genome stability regulators ATM and p53 further expand therapeutic targeting options. The dependency of ATM‐deficient tumour cells on PRKDC ubiquitination status offers novel strategies for chemotherapy sensitisation [26, 27]. p53 homeostasis regulation exhibits a sophisticated dynamic equilibrium: E3 ligases such as RNF115 and MDM2 promote its degradation, whereas deubiquitinases USP11 and USP7 enhance its stability [28, 29]. Importantly, combined USP7 inhibition and USP22 blockade synergistically activate p53 pathways [30], and TP53 mutation status significantly influences therapeutic responses to E3 ligases like TRIM65 [31, 32, 33], underscoring the necessity for personalised treatment strategies.

**FIGURE 2:**
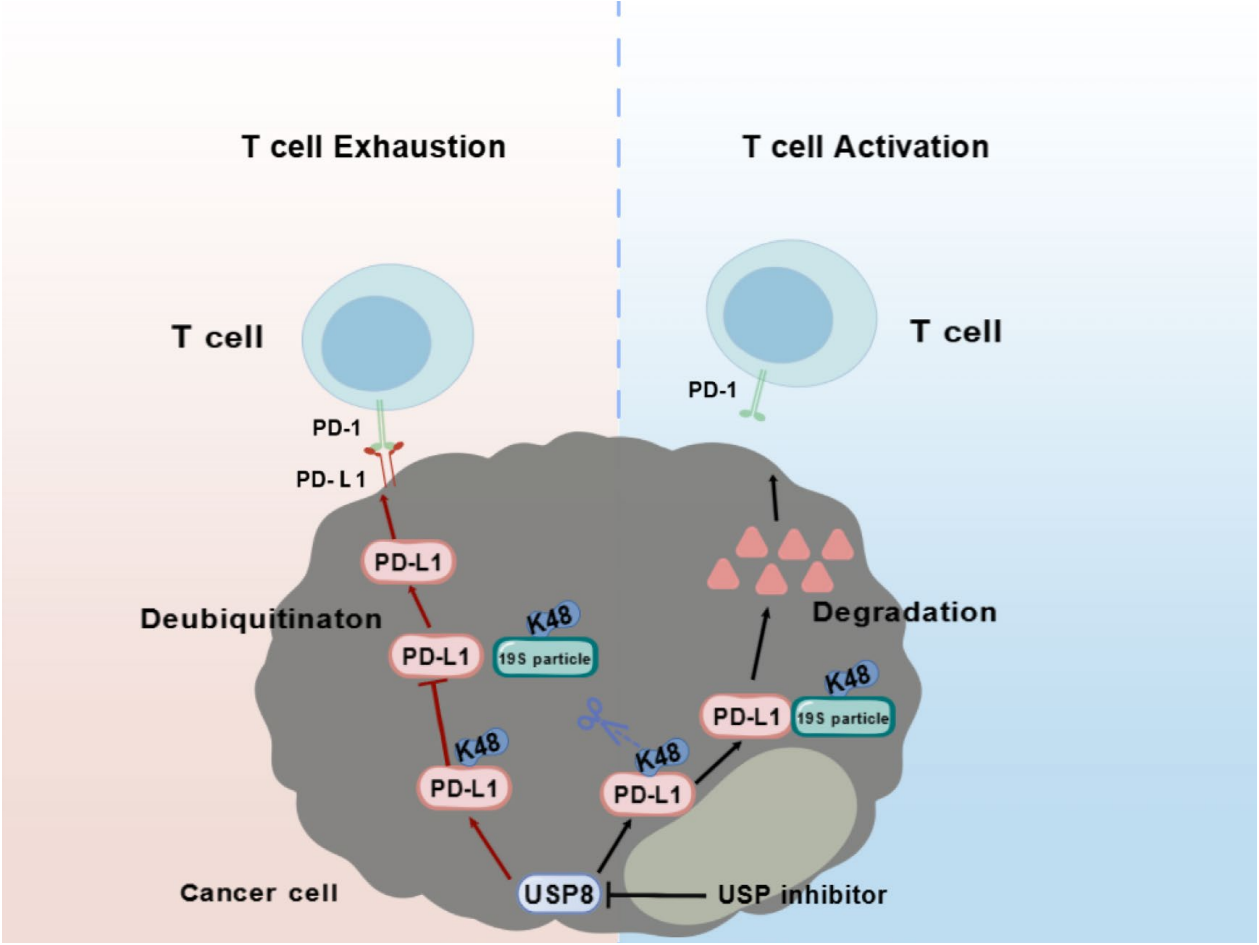
Mechanism of USP8‐mediated PD‐L1 Deubiquitination and its inhibition in cancer cells. This figure illustrates the dual mechanisms involving USP8 and PD‐L1 in cancer cells. On the left, it depicts how cancer cells utilise USP8 to deubiquitinate PD‐L1, thereby inhibiting its proteasomal degradation and mediating T cell exhaustion. On the right, it shows the schematic of specifically targeting and inhibiting USP8, which restores PD‐L1 ubiquitination and promotes its degradation, thereby reversing exhausted T cells into activated T cells and enhancing immune function.

PD‐L1, a pivotal immune checkpoint molecule, undergoes precise stability control by USP family members. USP8 inhibitors reverse T‐cell exhaustion by inducing PD‐L1 ubiquitination and degradation [34], whereas USP2/USP5 stabilise PD‐L1 to facilitate immune evasion [35, 36]. This bidirectional regulation implies that spatiotemporal‐specific inhibition of particular USPs may achieve dual benefits of “immune microenvironment remodeling” and “oncogenic signaling suppression.” For instance, USP8 inhibition not only reduces PD‐L1 levels but also enhances EGFR degradation through shared ubiquitination machinery, establishing a molecular rationale for combination therapies(Figure 2).

The maintenance of cancer stem cell (CSC) properties relies on specialised ubiquitination architectures. The PKMYT1AR/USP5 axis activates Wnt signalling by stabilising β‐catenin [37, 38], while FBP1 suppresses Notch signalling via NICD1 ubiquitination‐mediated degradation [39]. The USP14‐Vimentin interaction unveils a novel ubiquitination mechanism regulating epithelial‐mesenchymal transition (EMT) [40], and the ATG5‐p53‐autophagy axis directly links protein degradation to CSC maintenance [41]. These discoveries suggest that concurrent targeting of USP5 (β‐catenin) and USP14 (Vimentin) may cooperatively inhibit CSC self‐renewal and metastatic potential.

Despite the abundance of therapeutic targets within ubiquitination networks, clinical translation faces multifaceted challenges. Co‐occurring driver mutations (e.g., EGFR/KRAS) may activate compensatory ubiquitination pathways, while TP53 mutational heterogeneity demands precise therapeutic stratification [30, 31, 32, 33, 42]. Furthermore, developing tissue‐specific UPP modulators and biomarkers for ubiquitination network activity will be critical future directions. Integrating UPP‐targeted therapies with immune checkpoint inhibitors and epigenetic modulators may overcome current therapeutic limitations, advancing precision medicine for NSCLC patients.

## 3 Preclinical Insights Into Ubiquitination in NSCLC

### 3.1 The Impact of Ubiquitination on NSCLC Treatment Drugs

The importance of ubiquitination in the treatment of NSCLC is becoming increasingly evident, with several strategies targeting this mechanism showing potential to enhance anti‐tumour efficacy and overcome drug resistance. For example, by modulating the ubiquitination processes of molecules such as histone demethylase LSD1, PD‐L1 and RBM38, it is possible not only to promote immune cell infiltration but also to suppress tumour progression at the molecular level. Further exploration of the profound impact of ubiquitination on NSCLC treatment will provide new insights and potential targets for novel therapeutic strategies. Lysine‐specific histone demethylase 1A (LSD1) is an enzyme that alters the methylation status of histones and can promote the proliferation and progression of tumour cells, including those in lung cancer. Trim35 enhances immune cell infiltration and improves the anti‐tumour immunity of NSCLC by ubiquitinating LSD1 [43]. LINC02418, a negative regulator of PD‐L1 expression, can predict the prognosis of NSCLC patients. It downregulates PD‐L1 expression by enhancing the ubiquitination of PD‐L1 mediated by the E3 ligase Trim21, thereby improving the effectiveness of NSCLC treatment with anti‐PD‐L1 therapy [44]. Meanwhile, Kelch‐like ECH‐associated protein 1 (KEAP1), an E3 ligase, reduces PD‐L1 expression by facilitating its ubiquitination and proteasomal degradation, increasing CD8+ T cell infiltration and enhancing immune efficacy [45]. TRIM17 drives NSCLC resistance to cisplatin therapy by mediating the ubiquitination of RBM38 [46]. In addition, some members of the TRIM family regulate multiple signalling pathways (p53, NF‐κB, AKT, MAPK, Wnt/β‐catenin) and mediate the ubiquitination and proteasomal degradation of proteins, thereby promoting cancer proliferation, metastasis and resistance to cancer therapy. Long non‐coding RNAs (lncRNAs), a class of non‐coding RNAs longer than 200 nt, play a crucial role in drug resistance during NSCLC treatment with EGFR tyrosine kinase inhibitors (TKIs) [47, 48, 49]. LncRNAs LCETRL3 and LCETRL4 are highly expressed in NSCLC samples and significantly promote malignant proliferation. LCETRL3 inhibits TDP43 degradation via the UPP and activates the NOTCH1‐PTEN‐AKT signalling, leading to AKT signalling activation in NSCLC cells and contributing to TKI resistance [50]. Ubiquitin‐conjugating enzyme E2 variant 2 (UBE2V2) shows a positive correlation with PD‐L1 expression in NSCLC lung adenocarcinomas, suggesting poor efficacy of immune checkpoint inhibitors and susceptibility to resistance [51]. CRL2 KLHDC3, an E3 ligase complex where CRL2 is a subtype of the Cullin‐RING E‐type, and KLHDC3 is a subunit of the complex, is associated with poor overall survival in NSCLC patients. Researchers also suggest that NSCLC cell lines resistant to gefitinib exhibit higher levels of KLHDC3 protein [52]. In‐depth studies on how ubiquitination influences cancer cell growth and drug resistance through the regulation of multiple signalling pathways and protein degradation mechanisms could provide new insights and potential targets for the development and optimisation of NSCLC therapeutics.

### 3.2 In Vitro Studies Targeting Ubiquitination

Inhibiting the UPS to control the degradation of target substrate proteins is one of the therapeutic strategies for cancer treatment. Human cells contain over 100 deubiquitinases (DUBs) [15], which are classified into five distinct families: ubiquitin‐specific proteases (USPs), ubiquitin C‐terminal hydrolases (UCHs) [53], Machado‐Joseph domain‐containing family (MJDs), Josephin domain‐containing proteins [54] and ovarian tumour proteases (OTUs). Amongst these, the USP family is the largest and most extensively studied due to its structural and functional diversity, as well as its crucial role in the occurrence and development of various diseases [55]. USP5, a significant member of the USP family, plays a key role in regulating the interaction between E3 ubiquitin ligases and their substrate proteins by deubiquitinating and stabilising these proteins.

PROTACs represent an innovative therapeutic approach for targeted protein degradation. PROTACs utilise the cell's inherent protein degradation machinery to specifically degrade target proteins through small molecule compounds. This strategy has been applied in the treatment of various cancers, such as prostate cancer and triple‐negative breast cancer, particularly in hematologic malignancies [56, 57, 58]. EOAI 3402143 (G9) is a small molecule inhibitor of USP5 used in the treatment of NSCLC [59]. Its mechanism of action includes modulating USP5 to enhance the ubiquitination of Cyclin D1 (CCND1), leading to a reduction in its expression levels and exhibiting a synergistic effect with paclitaxel in lung cancer treatment [59]. By inducing DNA damage, EOAI 3402143 (G9) significantly upregulates γ‐H2AX expression in NSCLC cells, which activates the cell cycle regulator p53, thereby triggering cell cycle arrest, apoptosis and promoting autophagy [60]. The ability of G9 to induce CCND1 degradation and inhibit NSCLC cell growth has been validated.

Brigatinib, an inhibitor targeting ALK, may also exert antitumor effects through the inhibition of USP5 activity. Research has demonstrated its efficacy in suppressing colorectal cancer, and further studies are required to evaluate its therapeutic potential in NSCLC treatment [61].

### 3.3 In Vivo Studies Targeting Ubiquitination

In NSCLC research, animal model experiments related to ubiquitination have provided crucial insights for identifying potential therapeutic targets. USP22 has been identified as a regulator of c‐Myc function in NSCLC [30]. USP22 interacts with the c‐Myc protein, inducing its deubiquitination and maintaining its stability. Conversely, RNA interference (RNAi)‐mediated knockdown of USP22 in NSCLC cell lines or genetic depletion of USP22 in mouse models disrupts c‐Myc stability, thereby inhibiting its function. Notably, USP22 is present at higher levels in NSCLC patients with poor prognosis, and its expression is significantly correlated with the transcriptional activity of c‐Myc. Moreover, in xenograft mouse models, targeting USP22 has been shown to significantly inhibit the growth, angiogenesis and metastasis of NSCLC xenografts, as well as markedly extend the survival of mice bearing metastatic cancers [62]. Meanwhile, OTU2, a deubiquitinating enzyme from the OTU protein family, is also being explored [63]. Preclinical studies are underway to evaluate the USP and OTU families as either monotherapies or in combination with existing chemotherapy regimens for NSCLC treatment. Other similar targets include USP10 [64], USP51 [65], USP12 [66], OTUB1 [67], as well as small ubiquitin‐like modifiers (SUMOs) or SUMOylation. In conclusion, preclinical research in NSCLC primarily focuses on USP5, USP7 and key signalling pathway kinase inhibitors. Other USP inhibitors, such as the proteasome inhibitor 20S, have demonstrated promising results in preclinical studies for other cancers but remain relatively underexplored in NSCLC [68]. These model experiments further highlight the therapeutic potential of deubiquitinating enzymes, such as those from the USP and OTU families, in NSCLC and provide scientific justification for developing monotherapies or combination chemotherapy strategies. On‐going exploration of USP inhibitors holds the potential to advance the clinical application of ubiquitination‐targeted therapies in NSCLC treatment.

## 4 Clinical Research on Ubiquitination Drugs: A New Hope for NSCLC

### 4.1 Research Progress on Ubiquitination‐Related Drugs in NSCLC

Bortezomib, originally used for the treatment of MM, has been investigated in a trial to show the inhibition in proliferation of NSCLC cells when combined with nelfinavir, a protease inhibitor used in HIV treatment [69]. Carfilzomib, a second‐generation proteasome inhibitor, has demonstrated superior efficacy compared to bortezomib [70]. Marizomib, a proteasome inhibitor, has shown anti‐tumour activity in NSCLC and, when combined with HDAC inhibitors, synergistically enhances cellular stress and promotes cancer cell apoptosis [71]. Although the therapeutic efficacy has not yet been conclusively determined, these findings strongly support the potential use of marizomib in combination with HDAC inhibitors for NSCLC treatment. MLN4924 (also known as Pevonedistat), an NEDD8‐activating enzyme (NAE) inhibitor, is a first‐in‐class small molecule compound targeting Cullin‐RING ligases (CRLs). It inhibits the process of neddylation by blocking NAE, thus preventing the activation of CRLs, which play a crucial role in the cell cycle, including the regulation of tumour suppressor protein degradation. A phase II clinical trial indicated that the combination of Pevonedistat and Docetaxel in patients with relapsed or refractory stage IV NSCLC showed some efficacy. A total of 31 patients were enrolled, with an objective response rate (ORR) of 22%, a median progression‐free survival (PFS) of 4.1 months, and a median overall survival (OS) of 13.2 months. The primary adverse effects were neutropenia and liver impairment, leading to one patient's withdrawal due to a severe increase in transaminase levels [72] (Table 1). In the context of lung cancer treatment, strategies to improve the targeting specificity of UPS inhibitors may be adjunctive tools to reduce adverse effects. Bioinformatics analysis can be used to identify DUBs that are significantly upregulated in lung cancer tissues. For instance, JOSD2, USP5 and PSMD14 are found to be upregulated in NSCLC. Additionally, the substrate proteins of these DUBs can be predicted. In addition, the development of dual‐target drugs such as USP combined with KRAS inhibitors, precise delivery of nanomaterials, etc., may become improved strategies to increase drug specificity and reduce side effects in the future.

**TABLE 1:** Published clinical trial of ubiquitination‐related drugs in pan‐cancer in 2016–2024.

Clinical trial identifier	Phase	Results reporting dates	Cancer type (population, *N*)	Target	Interventions and combination	Outcomes
NCT00798720	II	28th April 2016	Advanced NSCLCr, *N* = 18	26S proteasome	Vorinostat + Bortezomib	Response time: 2 years; mOS: 5 years; Toxicity: 30 days post‐treatment.
NCT00389805	I/II	20th January 2017	Advanced NSCLCr or other solid tumours, *N* = 27	26S proteasome	Bortezomib + Pemetrexed disodium	Maximum tolerated dose: Arm A‐1.3 mg/m^2^; Arm B‐1.6 mg/m^2^
NCT04163107	I	7th January 2020	R/R‐MM, *N* = 19	26S proteasome	Hydroxychloroquine and carfilzomib	No results posted
NCT02512926	I	28th August 2021	Relapsed/refractory solid tumours or leukaemia, *N* = 4	26S proteasome	Carfilzomib in combination with Cyclophosphamide and etoposide	No results posted
NCT03944057	II	18th April 2023	Relapsed refractory multiple myeloma, *N* = 82	Exportin‐1 (XPO1)	ATG‐010	SAEs: 54.88%; all‐cause mortality: 8.54%
NCT03439293	II	15th June 2023	Relapsed refractory multiple myeloma, *N* = 82	26S proteasome	ATG‐010 80 mg plus Dexamethasone 20 mg	ORR: 12 months; PFS: 12 months; Serious AEs: 54.88%; all‐cause mortality: 8.54%.
NCT03323151	I/II	30th August 2023	Relapsed/refractory mantle cell lymphoma, *N* = 43	26S proteasome	Ixazomib + Ibrutinib	Ixazomib + BTK‐naïve: CR: 40.7%; ORR: 66.7%; PFS: 48 months; OS: 48 months.
NCT03345095	III	8th December 2023	Newly diagnosed glioblastoma, *N* = 749	26S proteasome	Experimental arm: Radiotherapy + Temozolomide + Marizomib followed by adjuvant Temozolomide + Marizomib, Standard arm: Radiotherapy + Temozolomide followed by adjuvant Temozolomide	OS: 49 months; PFS: 49 months; all‐cause mortality:experimental arm‐17.80%; standard arm‐18.57%; SAEs: experimental arm‐37.38%; standard arm‐26.94%

Abbreviations: AEs, adverse events; NSCLC, non‐small cell lung cancer; ORR, overall response; OS, overall response; PFS, Progression free survival; SAEs, serious adverse events.

### 4.2 The Application of PROTACs in Lung Cancer

In the field of lung cancer research, PROTACs (Proteolysis Targeting Chimeras) have emerged as a novel targeted therapy. Currently, validated therapeutic targets include EGFR, KRAS and ALK [73]. Traditional EGFR inhibitors, such as EGFR‐TKIs, face resistance issues during long‐term use, including mutations like T790M and C797S, which significantly reduce the long‐term efficacy of the drugs. The first EGFR‐targeted PROTACs were reported in 2018, utilising small molecules such as gefitinib and afatinib as ligands to bind to mutant EGFR, while recruiting E3 ligases like VHL. These molecules demonstrated selective degradation of mutant EGFR in cellular models, overcoming resistance to earlier inhibitors [74]. In 2020, the first KRAS G12C‐targeted PROTAC was reported by Gray et al. [75]. This PROTAC utilised ARS‐1620 to bind KRAS G12C and recruited the CRBN E3 ligase via a thalidomide derivative. However, the PROTAC showed limited effectiveness in degrading endogenous KRAS G12C. A VHL‐based PROTAC, utilising MRTX849 as a covalent ligand for KRAS G12C, was subsequently developed, which was demonstrated to induce KRAS degradation and inhibit MAPK signalling in various KRAS G12C‐mutant lung cancer cell lines, although its anti‐proliferative effects were not significantly enhanced compared to MRTX849 alone [76]. Another team developed the first agonist‐based SOS1 PROTAC, which demonstrated excellent anti‐proliferative activity in various KRAS‐driven cancer cells and showed anti‐tumour efficacy in the H358 xenograft model [77]. Although PROTACs have shown promise in in vitro and preclinical models, they have yet to undergo extensive validation in in vivo models. Future research will focus on optimising the pharmacokinetic properties of these PROTACs and evaluating their safety and efficacy in clinical trials.

### 4.3 The Application of Ubiquitination‐Related Drugs in Other Cancers

In 2003, bortezomib was approved by the FDA for the treatment of multiple myeloma (MM). Bortezomib inhibits the activation of the transcription factor nuclear factor kappa B (NF‐κB), thereby suppressing the proliferation and survival of MM cells. The activation of NF‐κB in MM cells is associated with drug resistance. Additionally, bortezomib enhances the sensitivity of MM cells to chemotherapy by interfering with the interactions between MM cells and the bone marrow microenvironment. Clinical trials have demonstrated its unique anti‐tumour activity against MM cells, with minimal effects on normal bone marrow or peripheral blood cells [78]. The use of bortezomib has since been extended to the treatment of NSCLC and pancreatic cancer in clinical trials [79]. However, its application is limited by off‐target effects and adverse reactions such as thrombocytopenia, peripheral neuropathy and gastrointestinal symptoms [80].

Nutlin, a small molecule MDM2 inhibitor, has been shown to be effective in treating hematologic malignancies, glioblastomas and AML cell growth [81, 82, 83]. Its mechanism of action involves inhibiting the interaction between MDM2 and p53, thereby increasing p53 stability and activity, which in turn suppresses tumour growth and metastasis. However, Nutlin is only effective in tumour cells with wild‐type p53 and is ineffective against tumours with p53 deletion or mutations [84].

VLX1570 is a small molecule DUB inhibitor targeting the 19S regulatory proteasome subunit. In a phase I clinical trial involving 15 patients, 14 received VLX1570 treatment. The results indicated that at dosages of 0.60 mg/kg and 1.2 mg/kg, VLX1570 exhibited anti‐tumour activity against MM. However, several patients developed severe pulmonary toxicity, with two fatalities, leading to premature termination of the study. These findings suggest that although VLX1570 has potential anti‐tumour activity, its severe pulmonary toxicity significantly limits its clinical application prospects [85]. To date, a series of clinical trials are underway in the field of pan‐cancer, including NSCLC, treatment to demonstrate its efficacy and safety (Table 2).

**TABLE 2:** On‐going clinical trials of ubiquitination‐targeted therapies across pan‐cancer from 2018 to 2024.

Clinical trial identifier	Phase	Start date	Status	Cancer type (population, N)	Target	Interventions and combination	Primary outcome measures	Secondary outcome measures
NCT03374085	I/II	6th February 2018	Active, not recruiting	R/R‐MM, *N* = 201	E3	CC‐92480 Monotherapy ± Dexamethasone	AEs, MTD, OR	PFS, OS
NCT04764942	I/II	6th May 2021	Recruiting	R/R‐MM, *N* = 81	Exportin‐1 (XPO1)	Selinexor + pomalidomide + dexamethasone ± carfilzomib.	Maximum tolerated dose, response rate	Pharmacokinetics activity, safety and tolerability, OS, PFS
NCT05675449	I	14th December 2022	Recruiting	R/R‐MM, *N* = 90	26S proteasome	Elranatamab + Carfilzomib + Dexamethasone/Maplirpacept	DLT	TEAE, AEs, BOR, ORR, PFS
NCT05654623	III	3th March 2023	Recruiting	Advanced metastatic breast cancer, *N* = 560	E3	ARV‐471 (PF‐07850327, vepdegestrant)/fulvestrant (FUL)	PFS	OS, ORR
NCT06050512	I/II	2th October 2023	Recruiting	R/R‐MM, *N* = 34	26S proteasome	Mezigdomide(5 mg/10 mg/15 mg) + Ixazomib + Dexamethasone	RP2D, ORR	AEs, DOR, PFS
NCT06536413	I/II	29th July 2024	Recruiting	Plasma cell myeloma patients, *N* = 42	26S proteasome	ATRA + Carfilzomib	RP2D	Efficacy
NCT02211755	I	6th October 2024	Recruiting	Refractory solid tumours, lymphomas, or myelodysplastic syndromes, *N* = 75	26S proteasome	Bortezomib + Clofarabine	Safety and MTD	ctDNA

Abbreviations: AEs, adverse events; BOR, best overall response; DLT, dose limiting toxicity; DOR, duration of response; MTD, maximum tolerated dose; ND, newly diagnosed; OR, overall response; ORR, objective response rate; OS, overall survival; PFS, progression free survival; RP2D, recommended phase ii dose; R/R‐MM, relapsed and refractory multiple myeloma; TEAE, treatment emergent adverse events; VGPR, percentage of participants with very good partial response.

## 5 Summary

This review explores the role of the UPS in the initiation, progression and treatment of NSCLC, emphasising the critical significance of ubiquitination as a vital post‐translational modification in regulating the stability, localisation and function of key proteins. The article also highlights the therapeutic potential of targeting DUBs in the treatment of lung cancer. By thoroughly examining the biological relevance of the UPS in NSCLC, current preclinical studies and its promise as an innovative therapeutic approach, this review aims to offer novel insights and strategies for the future diversification of treatment modalities for lung cancer patients.

## Author Contributions

Lianmin Zhang and Zhenfa Zhang contributed to the conception and design of the study; Jiaxiong Tan and Pengpeng Zhang collected and organised references; Guangyao Zhou wrote the first draft of the manuscript. All authors participated in the revision of the manuscript.

## Ethics Statement

The authors have nothing to report.

## Consent

The authors have nothing to report.

## Conflicts of Interest

The authors declare no conflicts of interest.

## Data Availability

Data sharing is not applicable to this article as no new data were created or analyzed in this study.
